# Correlation investigation between a single nucleotide polymorphism in ADAMTS14 (rs4747096) and osteoarthritis: a meta-analysis

**DOI:** 10.1186/s13018-023-04056-1

**Published:** 2023-08-07

**Authors:** Baojie Li, Xiaojing Li, Linjing Zhang, Leming Mou

**Affiliations:** Department of Orthopedics, The People’s Hospital of Weifang, 151 Guangwen Street, Kuiwen District, Weifang, 261000 Shandong China

**Keywords:** Osteoarthritis, Single nucleotide polymorphism, ADAMTS14, rs4747096, Susceptibility, Meta-analysis

## Abstract

**Background:**

Current evidence of the association between a single nucleotide polymorphism (SNP) in ADAMTS14 (rs4747096) and osteoarthritis (OA) is controversial. This study aimed to determine whether the ADAMTS14 SNP is closely related to OA risk.

**Methods:**

An electronic search of for the association between the rs4747096 polymorphisms and OA was performed using four online databases (updated on September 10, 2022). The association between susceptibility to OA and rs4747096 polymorphism was evaluated in four genetic models: the allele (mutation [A] vs. wild type [G]), additive (AA vs. GG and AG vs. GG), recessive (AA vs. AG + GG), and dominant (AA + AG vs. GG). This meta-analysis was performed in the R software, and effects were assessed using odds ratios (ORs) and 95% confidence intervals (CI).

**Results:**

Four studies (707 cases in the case group and 859 cases in the control group) were included. The results of the meta-analysis showed that, except in the recessive genetic model, there was a significant correlation between OA risk and the rs4747096 polymorphism using the allele (OR [95% CI] = 1.48 [1.26–1.73], *P* < 0.001), additive (AG vs. GG, OR [95% CI] = 2.56 [1.79–3.65], *P* < 0.001; AA vs. GG, OR [95% CI] = 2.81 [1.98–3.98], *P* < 0.001), and dominant (OR [95% CI)] = 1.72 [1.34–2.2], *P* < 0.001) genetic models.

**Conclusions:**

The ADAMTS14 rs4747096 polymorphism is associated with susceptibility to OA.

## Background

Osteoarthritis (OA), a common form of arthritis, contributes to disability and impairs quality of life [1]. Approximately 250 million people worldwide are affected by OA, accounting for 3.6% of the world’s population [2]. Although previous gene expression and associated biological function analyses have revealed aspects of OA development [3], the detailed molecular mechanism of the disease is not completely understood.

Single nucleotide polymorphisms (SNPs), the most common and stable variations, account for ~ 90% of gene polymorphisms in humans [4]. Several lines of evidence have determined the relationship between gene polymorphisms and orthopedic disorders and conditions [5–7]. As reported earlier, genetic factors combined with the environment and lifestyle play a key role in tendon and ligament injuries [8]. Previous studies have indicated that specific genomic variants can be used to predict the athletic performance of soccer players [9–11]. Clos et al. suggested that ACTN3 polymorphisms are associated with physical capability and tissue quality in professional football players [12]. The SNP in the elastin gene has been suggested as a predictive biomarker for ligament injury in football players [13, 14]. SNPs in the hepatocyte growth factor (HGF) gene have been confirmed to be associated with muscle injury [7].

ADAMTS14 is a member of the ADAMTS (a disintegrin-like and metalloproteinase domain with thrombospondin type 1 modules) metalloproteinase family, which is associated with knee OA [15]. The rs4747096 SNP in the *ADAMTS14* gene is closely related to knee OA susceptibility in the Chinese Han population [15]. ADAMTS14 is implicated in a variety of common biological conditions such as tumors and arthritis [16]. It can induce decreases in joint cartilage mechanical strength and further contribute to the development of OA [17]. Based on a case–control study, Mostafa et al. demonstrated an association between primary OA and *ADAMTS14* SNP rs4747096 [18]. Rodriguez-Lopez et al. showed that the G frequency of the nonsynonymous (cSNP) allele of the *ADAMTS14* gene rs4747096 was significantly higher in women with OA who required knee replacement than in the control group, further indicating that the ADAMTS14 polymorphism is closely related to knee OA [19]. In contrast, Haberal et al. did not identify any association between ADAMTS14 (rs4747096) genotypes and advanced-stage knee OA [20]. Thus, the correlation between the ADAMTS14 rs4747096 polymorphism and OA remains inconsistent. Meta-analysis refers to a statistical procedure that integrates the results of several independent studies that are considered combinable [21]. To obtain more comprehensive and objective results, the association (odds ratio [OR] and 95% confidence intervals [CI]) between OA susceptibility and the ADAMTS14 rs4747096 polymorphism was revealed using different genetic models, based on an updated meta-analysis. This study provides novel insights into the clinical prognosis of OA.

## Methods

### Data sources

**Fig. 1:**
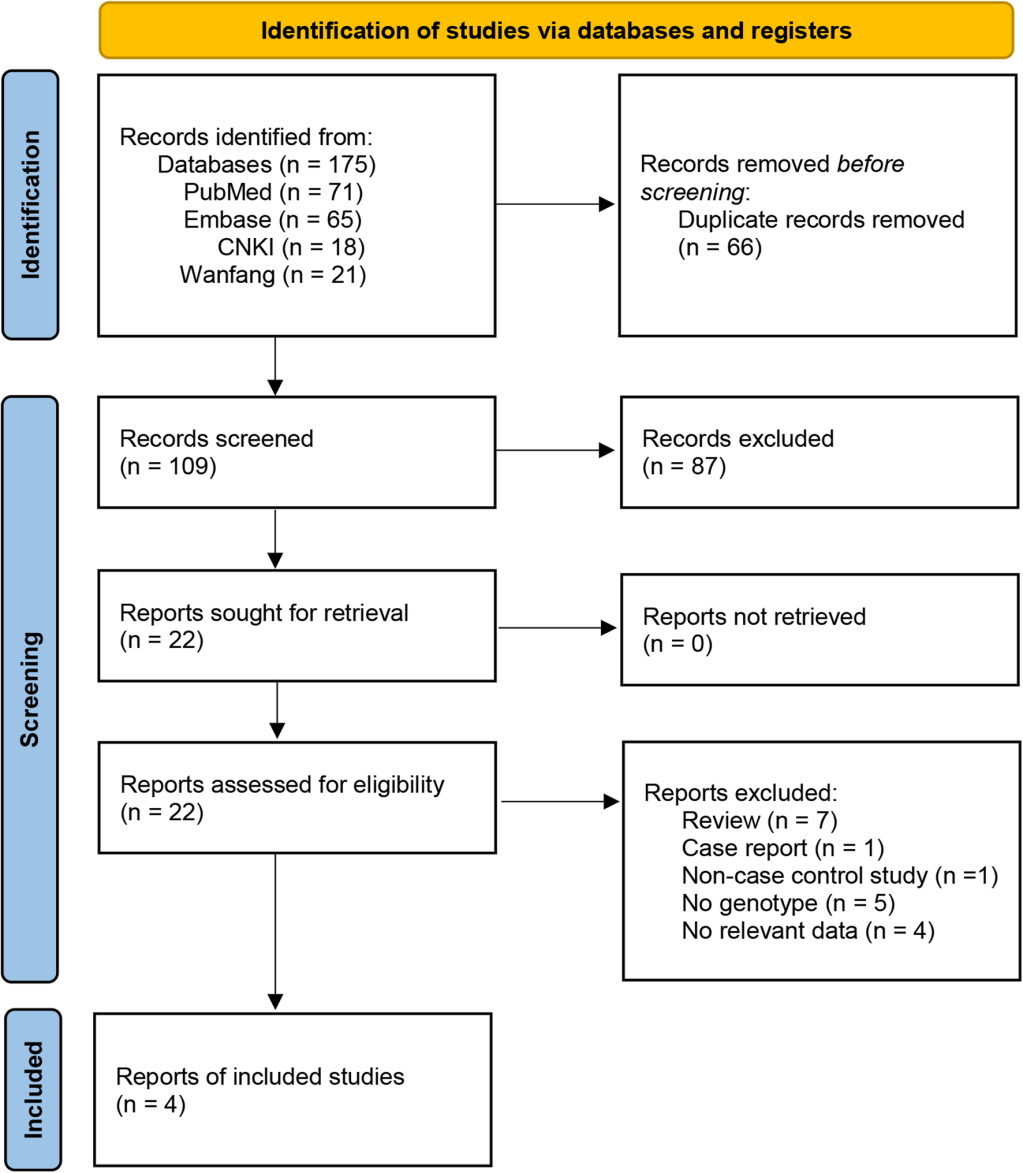
Flow diagram of the Preferred Reporting Items for Systematic Reviews and Meta-Analyses (PRISMA)

The search was based on the Preferred Reporting Items for Systematic Reviews and Meta-Analyses (PRISMA) guidelines. Relevant studies were searched from electronic databases, including PubMed, Embase, Wanfang Data, and CNKI. The main searching keywords included: “osteoarthritis” OR “ostarthritis” OR “ostearthritis,” “ADAMTS14” OR “rs4747096,” and “polymorphism” OR “mutation.” The search steps and corresponding results for each database are shown in Fig. 1. The deadline for document retrieval was updated to July 2, 2022. There were no language restrictions for document retrieval.

### Inclusion and exclusion criteria

The inclusion criteria for selecting studies comprised: (i) case–control studies with case (patients with OA) and control groups (healthy individuals); (ii) published literature on the relationship between the ADAMTS14 rs4747096 polymorphism and OA; and iii) provision or calculation of the number of genotypes/alleles in both the case and control groups. Reviews, reports, comments, and letters were excluded.

### Data extraction

Two researchers independently extracted articles according to the selection criteria. Next, the following data were independently collected from each eligible study: first author, year of publication, country, research time, gold standard of diagnosis, gene detection method, number of genotype distribution in the case and control groups, and general demographic data characteristics (such as sex ratio and age composition). If the extracted data were different between the two researchers, a consensus was reached through group discussion with a third scientist.

### Quality assessment

The quality assessment of all included studies was performed using the Newcastle–Ottawa Scale (NOS) recommended by the Agency for Healthcare Research and Quality (AHRQ) [22]. According to NOS, the quality of the methodology in each study was explored from three aspects: selected subjects (4 points), comparability (2 points), and exposure (3 points). Specifically, high-, medium-, and low-quality studies were scored 7–9, 4–6, and < 4 points, respectively.

### Statistical analysis

Evaluation of the Hardy–Weinberg equilibrium (HWE) was performed on the population in the control group using the chi-square test [23], and the studies that did not conform to the HWE test (*P* < 0.05) were excluded [24]. The R software (version 3.10) was used for statistical analysis. The meta-analysis in the current study was performed on the association between OA susceptibility and the genetic polymorphism of rs4747096 using four genetic models: the allele (mutation [A] vs. wild type [G]), additive (AA vs. GG and AG vs. GG), recessive (AA vs. AG + GG), and dominant (AA + AG vs. GG). Significant differences between groups were evaluated using 95% confidence intervals (CI). Heterogeneity among the included studies was revealed based on Cochran’s *Q* test and *I*^2^ [25]. If heterogeneity was observed, a random-effects model was used (*P* < 0.05 or *I*^2^ > 50%); otherwise, a fixed-effects model was applied (*P* ≥ 0.05 or *I*^2^ ≤ 50%). Moreover, sensitivity analysis and the Egger’s test were performed to investigate the stability and publication bias in the combined results, respectively [26].

## Results

### Suitable article exploration

A total of 175 studies were extracted based on all searched databases. After eliminating the duplicate literature, 109 studies were retained. Of these, 87 articles were excluded after reading titles and abstracts. Of the remaining 22 studies, 18, including literature reviews, case series, non-case–control studies, and studies that did not describe specific genotypes, were excluded after full-text reading. Finally, four studies [15, 17, 20, 27] with sufficient data were included in this meta-analysis (Fig. 1).

### Information on included studies

A total of 1566 cases were included: 707 cases in the case group (382 men and 325 women) and 859 cases (449 men and 410 women) in the control group. The publication years of the four studies ranged from 2013 to 2021 and the ages of the patients from 31 to 70 years. There were no significant differences in age or sex between the two groups. The study locations were China, Turkey, and Thailand. OA detection mainly included RDC/TMD, ACR, and the Kellgren–Lawrence grading system. The quality evaluation showed that the quality of the included studies was ideal (NOS score: 6–8). The detailed characteristics of the four studies are listed in Table 1. Furthermore, the distribution results of the rs4747096 genotypes in each study showed that the control group in the selected literature obeyed the HWE test, confirming that the population selected was representative (Table 2).

**Table 1:** Characteristics of four enrolled studies in current meta-analysis

Items	Studies			
Author	Wang^1^	Haberal^2^	Ma^3^	Poonpet^4^
Public year	2018	2021	2018	2013
Location	China	Turkey	China	Thailand
Study year	NA	2018.10–2019.03	2013–2017	NA
Detection method	NA	RT-PCR	PCR–RFLP	PCR–RFLP
Diagnostic criteria	RDC/TMD	ACR	Kellgren–Lawrence grading system	Kellgren–Lawrence grading system
Type	TMJOA	Advanced-stage KOA	KOA	KOA
Score*	6	8	7	8
Case subjects				
Male/female	0/103	124/26	170/176	88/20
Age (years, mean ± SD)	33.7 ± 13.4	66 ± 9.8	57.1 ± 7.0	70 (51–91)
Control subjects				
Male/female	0/110	108/42	248/232	93/26
Age (years, mean ± SD)	30.9 ± 9.6	57.3 ± 11.2	56.6 ± 7.0	55 (50–60)

*NOS Score, The Newcastle–Ottawa Scale; KOA, knee osteoarthritis; TMJOA, temporomandibular joint osteoarthritis; ACR, American College of Rheumatology; RDC/TMD, research diagnostic criteria for temporomandibular disorders; PCR–RFLP, polymerase chain reaction-restriction fragment length polymorphism; and RT-PCR, real-time polymerase chain reaction

^1^Dan-dan, W., et al., *Association between ADAMTS14 gene polymorphism and the temporomandibular joint osteoarthritis in Chinese Han females.* Journal of Peking University (Health Sciences), 2018. **50**(2): p. 279–283

^2^Haberal, B., et al., *Lack of association between MMP13 (rs3819089), ADAM12 (rs3740199-rs1871054) and ADAMTS14 (rs4747096) genotypes and advanced-stage knee osteoarthritis.* Joint Diseases and Related Surgery, 2021. **32**(2): p. 299–305

^3^Ma, S., C. Ouyang, and S. Ren, *Relationship between ADAMTS14/rs4747096 gene polymorphism and knee osteoarthritis in Chinese population.* Bioscience Reports, 2018. **38**(5)

^4^T.Poonpet, et al., *ADAMTS14 gene polymorphism associated with knee osteoarthritis in Thai women.* Genetics and Molecular Research, 2013. **12**(4): p. 5301–5309

**Table 2:** The distribution results of rs4747096 genotype in each enrolled study

Author	Public year	Case subjects					Control subjects					HWE in control	
		AA	AG	GG	A	G	AA	AG	GG	A	G	chi-square	*P*
Wang DD	2018	40	57	6	137	69	45	48	17	138	82	0.4910	0.5367
Haberal B	2021	116	33	1	265	35	118	29	3	265	35	0.5765	0.6995
Ma S	2018	146	126	74	418	274	256	182	42	694	266	1.3760	0.2606
Poonpet T	2013	50	46	12	146	70	48	46	25	142	96	4.6131	0.0392

HWE, Hardy–Weinberg equilibrium, it was evaluated using the likelihood-ratio chi-square test, and *P* values were presented. *P* < 0.05 was considered representative of a departure from HWE

### Meta-analysis results

**Fig. 2:**
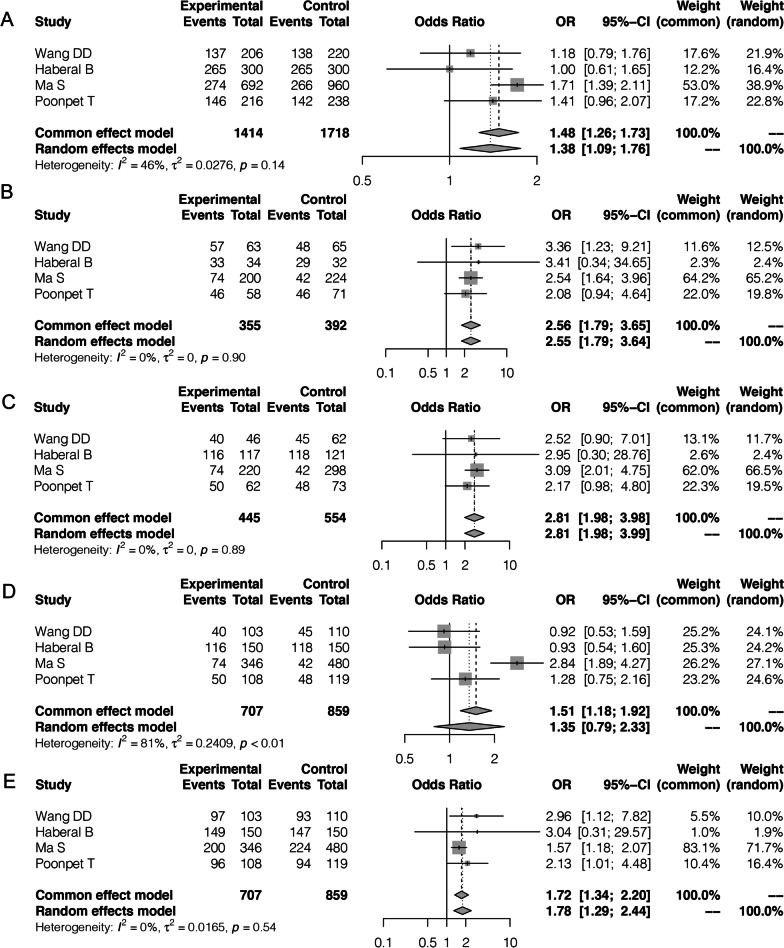
The meta-analysis revealed the association between osteoarthritis (OA) susceptibility and genetic polymorphism of rs4747096 using four genetic models. **A** Allele genetic model (mutation [A] vs. wild type [G]); **B** additive genetic model (AA vs. GG); **C** additive genetic model (AG vs. GG); **D** recessive genetic model (AA vs. AG + GG); and **E** dominant genetic model (AA + AG vs. GG)

The forest plot for the current analysis is shown in Fig. 2A–D and Table 3. Because heterogeneity was not significant (*P* = 0.14, *I*^2^ = 46%) among these studies in the allele genetic model (A vs. G), the fixed-effect model was adopted. The pooled results (OR = 1.48, 95% CI 1.26–1.73, *Z* = 4.87, *P* < 0.001) showed that there was a significant correlation between the rs17222919 polymorphism and OA risk in the allele genetic model (Fig. 2A). Moreover, because of the significant differences among these studies using the additive genetic model AG versus GG (*P* = 0.90, *I*^2^ = 0%) and AA versus GG (0.89, *I*^2^ = 0%), the fixed-effect model was used to pool results. The pooled results revealed that the rs4747096 polymorphism was significantly associated with increased risk of OA using the additive model (AG vs. GG, OR [95% CI] = 2.56 [1.79–3.65], *P* < 0.001; AA vs. GG, OR [95% CI] = 2.81 [1.98–3.98], *P* < 0.001) (Fig. 2B, C). In addition, the fixed-effect model was used to pool results because heterogeneity among these studies was not significant (*P* = 0.89, *I*^2^ = 0%) using the additive genetic model. The pooled results (OR = 2.81, 95% CI 1.98–3.98, *Z* = 5.77, *P* < 0.001) showed a significant correlation between rs4747096 polymorphism and OA risk using the additive genetic model (Fig. 2C). Furthermore, considering the lack of significant heterogeneity in the recessive genetic model (*P* < 0.01, *I*^2^ = 81%), the random-effects model was used. The pooled results showed that, compared with the AA genotype, rs4747096 variants (GG + AG) did not increase the risk of OA (OR = 1.35, 95% CI 0.79–2.339, *Z* = 1.09, *P* = 0.28). Finally, because the heterogeneity among these studies was not significant (*P* = 0.54, *I*^2^ = 0%) in the dominant genetic model (AA + AG vs.GG), the fixed-effects model was applied. The pooled results showed that the polymorphism of rs4747096 (GG) was a risk factor for OA compared with the AA/AG genotype combination (OR = 1.72, 95% CI 1.34–2.2, *Z* = 4.24, *P* < 0.001; Fig. 2E). Altogether, these results show that the ADAMTS14 rs4747096 polymorphism is associated with susceptibility to OA using the allele, additive, and dominant genetic models.

**Table 3:** Main meta-analysis results

Model	Subjects		Heterogeneity^a^	Statistical model	OR (95% CI)	Overall effect	Egger’s test^b^
	Cases	Control				*P* value	*P* value
A versus G	1414	1718	No	Fixed	1.4787 [1.2633; 1.7308]	< 0.001	0.0314
AG versus GG	355	392	No	Fixed	2.5579 [1.7916; 3.6517]	< 0.001	0.7457
AA versus GG	445	554	No	Fixed	2.8057 [1.9758; 3.9842]	< 0.001	0.4000
AA versus GG + AG	707	859	Yes	Random	1.3536 [0.7857; 2.3321]	0.2753	0.0077
AA + AG versus GG	707	859	No	Fixed	1.7157 [1.3371; 2.2015]	< 0.001	0.1035

^a^Random-effects model was used when there was heterogeneity; otherwise, the fixed-effect model was used

^b^Egger’s test to evaluate publication bias; *P* < 0.05 is considered statistically significant. OR, odds ratio; CI, confidence interval

### Sensitivity and publication bias analyses

**Fig. 3:**
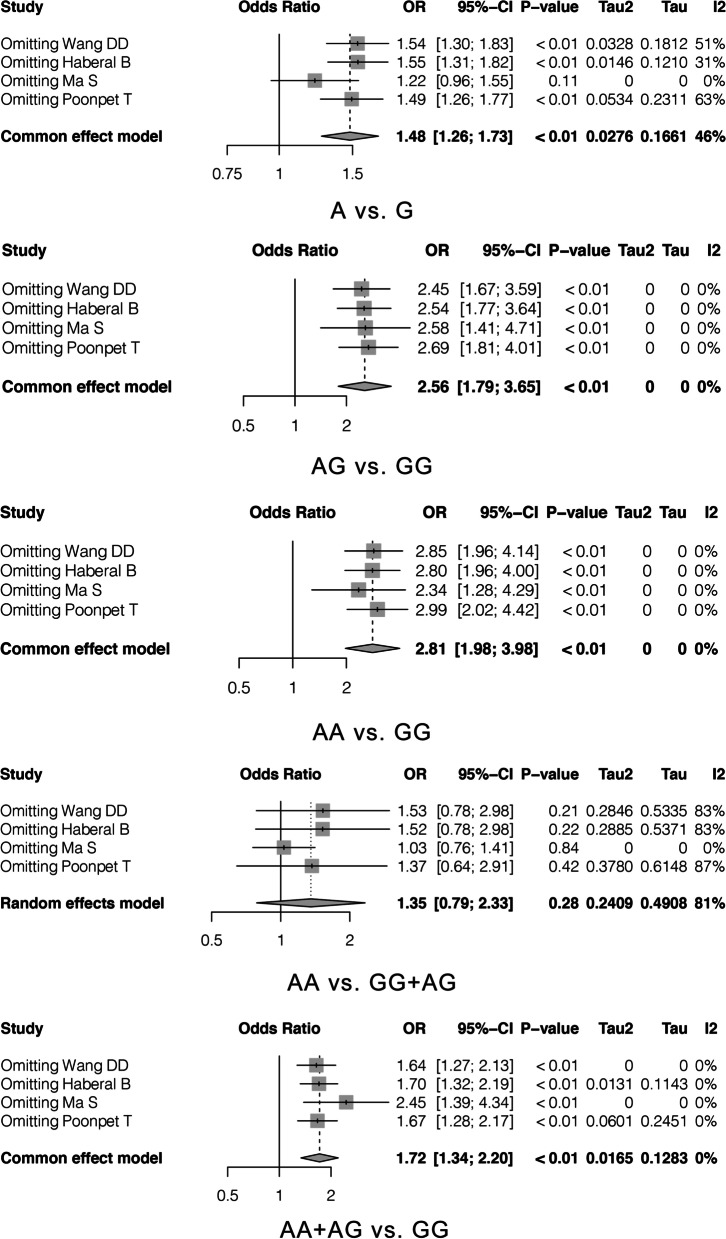
Sensitivity and publication bias analysis. **A** The result of sensitivity analysis. **B** The result of publication bias analysis

The results showed that the OR value in the allele model changed after removing the study by Ma et al. [15], while the OR values in the additive, recessive, and dominant genetic models were not affected, suggesting that the results were stable (Fig. 3). The Egger’s test showed that, except for the allele (*P* = 0.0314) and recessive (*P* = 0.0077) genetic models, no publication bias was observed in the other models (Table 3).

## Discussion

Although the association between ADAMTS14 SNPs with the progression of OA [28] has been confirmed, the detailed correlation between the ADAMTS14 rs4747096 polymorphism and OA susceptibility is still controversial. The current study indicated that the ADAMTS14 rs4747096 polymorphism in the allele, additive, and dominant genetic models significantly correlated with OA susceptibility.

ADAMTS proteins constitute a family of zinc metalloproteinases that target and process extracellular matrix proteins [29]. ADAMTS14, located on human chromosome 10q2, is a member of the ADAMTS metalloproteinase family [30]. Nonsynonymous polymorphisms, such as that of a major aggrecanase of the ADAMTS family genes, are known to confer susceptibility to OA [5]. A previous study has shown that the ADAMTS14 rs4747096 GG variant contributes to injury recovery in human Achilles tendon pathology [31]. It has been confirmed that the number of GG/AG combinations in the ADAMTS14/rs4747096 gene polymorphism is more common in patients with OA, and, compared with the AA genotype, GG, AG, and GG/AG significantly increase the risk of OA [15]. Wen et al. indicated that SNPs at loci (such as rs3171407, rs229071, and rs229077) of the *ADAMTS* gene are related to OA risk, and the underlying SNPs affect the regulation of the ADAMTS protein expression through miRNAs [32]. In this study, the ADAMTS14 rs4747096 polymorphism using the allele (A vs. G), additive (AG vs. GG and AA vs. GG), and dominant (AA + AG vs. GG) genetic models statistically correlated with OA susceptibility. Based on this study, we speculate that there is an association between the SNP rs4747096 of ADAMTS14 and OA, further indicating that the ADAMTS14 rs4747096 polymorphism is associated with OA susceptibility. Thus, the *ADAMTS14* gene SNP may be proposed as a biomarker for the diagnosis, prevention, and treatment of OA.

In this study, a meta-analysis was performed to assess the correlation between rs4747096 and susceptibility to OA, thereby rendering the conclusion more reliable. However, there are some limitations in the current study that cannot be ignored. Specifically, (i) there were some differences with regard to races and regions, differences in regional living habits and environments, sex, age, and other confounding factors among the different models; (ii) due to the incomplete data in some studies, the covariates, which may affect the results of the meta-analysis as potential confounders, were not corrected; (iii) in this study, only one locus of ADAMTS14 (rs4747096) was selected for analysis (other ADAMTS14 loci were not included in relevant eligible literature), which could lead to inappropriate judgment of the overall genetic effect on OA risk; (iv) the pathological type and clinical staging data of OA were not analyzed in current study due to incomplete data; (v) no subgroup analysis of age and race was performed, given that only four studies were included in this analysis; (vi) there was publication bias in the allele (A vs. G) and recessive (AA vs. GG + AG) genetic models; and (vii) the sensitivity analysis results showed that the allele genetic model (A vs. G) was not stable. Thus, these may arise due to the small size of samples and limited number of eligible studies.

## Conclusions

The ADAMTS14 rs4747096 polymorphism was significantly associated with susceptibility to OA using the allele, additive, and dominant genetic models. The rs4747096 polymorphism may be a candidate marker for the screening, diagnosis, and treatment of OA. A larger-scale, independent replicated association analysis or larger sample sizes of updated meta-analyses are needed to verify the authenticity of the results.

## Data Availability

The datasets generated and/or analyzed during the current study are not publicly available because the research project is not yet complete and the data remain confidential, but are available from the corresponding author on reasonable request.

## Abbreviations

SNP: Single nucleotide polymorphism
OA: Osteoarthritis
NOS: Newcastle–Ottawa Scale
OR: Odds ratio
PRISMA: Preferred Reporting Items for Systematic Reviews and Meta-Analyses

## References

[1] Maffulli N (2022). Osteoarthritis and the middle aged athlete: the present and future. Sports Med Arthrosc Rev.

[2] Prieto-Alhambra D, Barbero MH, Verges J (2013). SAT0322Trends of use of different Sysadoa drugs in patients with osteoarthritis from 2006 to 2011: a population-based cohort study. Osteoarthr Cartil.

[3] Johnson VL, Hunter DJ (2014). The epidemiology of osteoarthritis. Best Pract Res Clin Rheumatol.

[4] Barendse W, Bunch RJ, Thomas MB, Harrison BE (2010). A splice site single nucleotide polymorphism of the fatty acid binding protein 4 gene appears to be associated with intramuscular fat deposition in longissimus muscle in Australian cattle. Anim Genet.

[5] Rodriguez-Lopez J, Mustafa Z, Pombo-Suarez M, Malizos KN, Rego I, Blanco FJ (2014). Genetic variation including nonsynonymous polymorphisms of a major aggrecanase, ADAMTS-5, in susceptibility to osteoarthritis. Arthritis Rheum.

[6] Aicale R, Tarantino D, Maccauro G, Peretti GM, Maffulli N (2019). Genetics in orthopaedic practice. J Biol Regul Homeost Agents.

[7] Pruna R, Artells R, Lundblad M, Maffulli N (2017). Genetic biomarkers in non-contact muscle injuries in elite soccer players. Knee Surg Sports Traumatol Arthrosc.

[8] Longo UG, Loppini M, Margiotti K, Salvatore G, Berton A, Khan WS (2015). Unravelling the genetic susceptibility to develop ligament and tendon injuries. Curr Stem Cell Res Ther.

[9] Kambouris M, Del Buono A, Maffulli N (2014). Genomics DNA profiling in elite professional soccer players: a pilot study. Transl Med UniSa.

[10] Maffulli N, Margiotti K, Longo UG, Loppini M, Fazio VM, Denaro V (2013). The genetics of sports injuries and athletic performance. Muscles Ligaments Tendons J.

[11] Kambouris M, Ntalouka F, Ziogas G, Maffulli N (2012). Predictive genomics DNA profiling for athletic performance. Recent Pat DNA Gene Seq.

[12] Clos E, Pruna R, Lundblad M, Artells R, Maffulli N (2021). ACTN3's R577X single nucleotide polymorphism allele distribution differs significantly in professional football players according to their field position. Med Princ Pract.

[13] Artells R, Pruna R, Dellal A, Maffulli N (2016). Elastin: a possible genetic biomarker for more severe ligament injuries in elite soccer. A pilot study. Muscles Ligaments Tendons J.

[14] Pruna R, Artells R, Ribas J, Montoro B, Cos F, Muñoz C (2013). Single nucleotide polymorphisms associated with non-contact soft tissue injuries in elite professional soccer players: influence on degree of injury and recovery time. BMC Musculoskelet Disord.

[15] Ma S, Ouyang C, Ren S (2018). Relationship between ADAMTS14/rs4747096 gene polymorphism and knee osteoarthritis in Chinese population. Biosci Rep..

[16] Colige A, Vandenberghe I, Thiry M, Lambert CA, Beeumen JV, Li SW (2002). Cloning and characterization of ADAMTS-14, a novel ADAMTS displaying high homology with ADAMTS-2 and ADAMTS-3. J Biol Chem.

[17] Poonpet T, Honsawek S, Tammachote N, Kanitnate S, Tammachote R (2013). ADAMTS14 gene polymorphism associated with knee osteoarthritis in Thai women. Genet Mol Res.

[18] Mostafa N, Ibrahim IK, Mikhael NL, Saba E (2022). Association between primary osteoarthritis and ADAMTS14 single nucleotide polymorphism in Egyptian population: a case-control study. Egypt Rheumatol Rehabil.

[19] Gonzalez A, Rodriguez-Lopez J, Pombo-Suarez M, Mustafa Z, Loughlin J, Tsezou A (2007). 292 A change of glutamic acid for glicine in the ADAMTS14 protease associated with susceptibility to primary knee osteoarthritis. Osteoarthr Cartil.

[20] Haberal B, Imek EK, Tu Z, Cebi H, Atac FB (2021). Lack of association between MMP13 (rs3819089), ADAM12 (rs3740199-rs1871054) and ADAMTS14 (rs4747096) genotypes and advanced-stage knee osteoarthritis. Joint Dis Relat Surg.

[21] Borenstein M, Hedges LV, Higgins JP, Rothstein HR (2021). Introduction to meta-analysis.

[22] Wells G, Shea B, O'Connell D, Peterson J, Welch V, Losos M. The Newcastle–Ottawa Scale (NOS) for assessing the quality of nonrandomised studies in meta-analyses. http://www.ohri.ca/programs/clinical_epidemiology/oxford.asp.

[23] Schaid DJ, Jacobsen SJ (1999). Blased tests of association: comparisons of allele frequencies when departing from Hardy-Weinberg proportions. Am J Epidemiol.

[24] Zintzaras E, Lau J (2008). Synthesis of genetic association studies for pertinent gene–disease associations requires appropriate methodological and statistical approaches. J Clin Epidemiol.

[25] Higgins JP, Thompson SG, Deeks JJ, Altman DG (2003). Measuring inconsistency in meta-analyses. BMJ.

[26] Egger M, Davey Smith G, Schneider M, Minder C (1997). Bias in meta-analysis detected by a simple, graphical test. BMJ.

[27] Dan-dan W, Ye-hua G, Xu-chen M, Juan-hong M (2018). Association between ADAMTS14 gene polymorphism and the temporomandibular joint osteoarthritis in Chinese Han females. J Peking Univ Health Sci.

[28] Spector TD, Rodriguez-Lopez J, Slagboom PE, Valdes AM, Blanco FJ, Pombo-Suarez M (2009). Association of a nsSNP in ADAMTS14 to some osteoarthritis phenotypes. Osteoarthritis Cartilage..

[29] Pluda S, Mazzocato Y, Angelini A (2021). Peptide-based inhibitors of ADAM and ADAMTS metalloproteinases. Front Mol Biosci.

[30] Bolz H, Ramírez A, Brederlow BV, Kubisch C (2001). Characterization of ADAMTS14, a novel member of the ADAMTS metalloproteinase family. Biochim Biophys Acta.

[31] Khoury LE, Posthumus M, Collins M, Handley CJ, Cook J, Raleigh SM (2013). Polymorphic variation within the ADAMTS2, ADAMTS14, ADAMTS5, ADAM12 and TIMP2 genes and the risk of Achilles tendon pathology: a genetic association study. J Sci Med Sport.

[32] Weng K, Luo M, Dong D (2020). Elucidation of the mechanism by which a ADAMTS5 gene microRNA-binding site single nucleotide polymorphism affects the risk of osteoarthritis. Genet Test Mol Biomark.

